# 

**Published:** 1865-08

**Authors:** 


### CHICAGO MEDICAL JOURNAL.

**Terms, $2.00 per Annum.**

### CONTENTS OF THE AUGUST NUMBER.

PAGE.

ORIGINAL COMMUNICATIONS.

On the Treatment of Dysentery. By Dr. D. B. Trimble, of Chicago, Ill., 337

Five Cases of Military Surgery. By Israel B. Washburn, M. D., late Surgeon 46th Ind. Vol. Inf. .... 342

Chronic Diarrhœa. By George D. Winch, M.D., Surg. 42d Wis. Vol. Inf., 344

SELECTED.

Cork and Its Uses. By John R. Jackson .... 351

Submarine Telegraphs .... 378

EDITORIAL AND MISCELLANEOUS.

Dr. Brainard's Address before the American Dental Association .... 356

Acute Pleuro-Pneumonia, producing Empyemia and Consolidation of the Lungs. By E. R. Frances, M. D., of Amboy, Ill .... 369

Mechanical Treatment of Deformities. By C. R. Blackall, M. D., of Chicago, Ill .... 371

Chloroform .... 374

The American Dental Association .... 375

Proceedings of De Witt County Medical Society .... 375

Proceedings of Fox River Valley Medical Society .... 376

Graduates of Medical Colleges—Marriages, etc .... 384

BOOK NOTICES AND REVIEWS .... 382

